# The Dopamine Transporter Gene, a Spectrum of Most Common Risky Behaviors, and the Legal Status of the Behaviors

**DOI:** 10.1371/journal.pone.0009352

**Published:** 2010-02-22

**Authors:** Guang Guo, Tianji Cai, Rui Guo, Hongyu Wang, Kathleen Mullan Harris

**Affiliations:** 1 Department of Sociology, The University of North Carolina, Chapel Hill, North Carolina, United States of America; 2 Carolina Center for Genome Sciences, The University of North Carolina, Chapel Hill, North Carolina, United States of America; 3 Caroline Population Center, The University of North Carolina, Chapel Hill, North Carolina, United States of America; 4 School of Library and Information Sciences, North Carolina Central University, Durham, North Carolina, United States of America; 5 Department of Sociology, University of Macau, Av. Padre Tomás Pereira, Taipa, Macau; University of Muenster, Germany

## Abstract

This study tests the specific hypothesis that the 9R/9R genotype in the VNTR of the dopamine transporter gene (*DAT1*) exerts a general protective effect against a spectrum of risky behaviors in comparison to the 10R/9R and 10R/10R genotypes, drawing on three-time repeated measures of risky behaviors in adolescence and young adulthood on about 822 non-Hispanic white males from the Add Health study. Our data have established two empirical findings. The first is a protective main effect in the *DAT1* gene against risky behaviors. The second finding is that the protective effect varies over age, with the effect prominent at ages when a behavior is illegal and the effect largely vanished at ages when the behavior becomes legal or more socially tolerated. Both the protective main effect and the gene-lifecourse interaction effect are replicated across a spectrum of most common risky behaviors: delinquency, variety of sexual partners, binge drinking, drinking quantity, smoking quantity, smoking frequency, marijuana use, cocaine use, other illegal drug use, and seatbelt non-wearing. We also compared individuals with the protective genotype and individuals without it in terms of age, physical maturity, verbal IQ, GPA, received popularity, sent popularity, church attendance, two biological parents, and parental education. These comparisons indicate that the protective effect of DAT1*9R/9R cannot be explained away by these background characteristics. Our work demonstrates how legal/social contexts can enhance or reduce a genetic effect on risky behaviors.

## Introduction

The objective of this study is twofold: to provide credible evidence for a protective effect regarding the dopamine transporter gene (*DAT1*) and to show how the legal and social context may influence the strength of such an effect. Our first objective is to test if the 9R/9R genotype in the VNTR of the dopamine transporter gene (*DAT1*) has a protective effect against a spectrum of risky behaviors relative to the 9R/10R or 10R/10R genotype. Although previous work has examined the links between the *DAT1* gene and tobacco and alcohol consumption [1], [2], no work has examined the link with a large number of health behaviors simultaneously in one single study sample: delinquency (a collection of criminal behaviors), number of sexual partners, binge drinking, drinking quantity, smoking quantity, smoking frequency, marijuana use, cocaine use, other illegal drug use (LSD, PCP, ecstasy, mushrooms, speed, ice, heroin, or pills), and seatbelt non-wearing. Our second objective examines whether the strength of the protection effect interacts with the lifecourse in adolescence and adulthood in such a way that can be explained by the age-specific legal status of a behavior.

The Dopamine Transporter Gene (*DAT1*, locus symbol: *SLC6A3*) or the soluble carrier family 6 dopamine transporter member three gene, codes for a dopamine transporter protein (DAT), which limits the level and duration of dopamine receptor activation [3]. Decades of research have accumulated evidence for the integral role of dopaminergic neurotransmission in the regulation of additive and rewarding behaviors [4] and in memory and learning [5]. A number of animal studies have demonstrated that natural rewarding stimuli such as food, drink, and sex increase the *in-vivo* release of dopamine in the nucleus accumbens [6]. Vanderbergh et al. [7] identified a polymorphic 40-bp variable number of tandem repeats (VNTR) in the *DAT1* gene, which is most commonly observed repeat 9 (DAT1*9R) or 10 times (DAT1*10R). Although the VNTR is located in a section encoding the 3′ untranslated region and does not change the protein's amino acid sequence, it has been shown to have functional effects on gene expression. Please refer to Haddley et al. [8] for an excellent review on the dopamine transporter gene and addictive behaviors.

Functional and association studies involving the dopamine transporter gene is often characterized by inconsistency. For example, One in-vivo study [9] reported lower levels of *DAT1* expression in the striatal putamen for individuals with a DAT1*9R/10R genotype when compared to those homozygous for the 10R allele. However, other studies [10], [11], [12] reported opposite findings showing that higher levels of striatal *DAT1* availability in individuals with one or two 9R alleles. Three additional in-vivo studies found no effect of the VNTR polymorphism on *DAT1* density [13], protein availability [14] or function [14].

Quite a number of studies have attempted to link the *DAT1* VNTR with addictive or risky behaviors. A 9R allele has been linked with a lowered risk of smoking addiction [15], [16], [17]. Studies on *DAT1* and alcoholism mostly failed to demonstrate a link between the two [18], [19]. Many studies of the *DAT1* VNTR have examined ADHD and the findings are inconsistent. Several family transmission studies showed a higher prevalence in ADHD among individuals with a 10R allele [20], [21], [22], [23], [24], [25] while another study demonstrated an association of the 9R/10R genotype with more severe symptoms of ADHD in comparison to the 10R/10R genotype [26]. Still other family-based [27], [28] and population-based studies found no association between the VNTR and ADHD [27], [29]. Conflicting evidence may have arisen from a large number of factors such as the type of association studies (e.g., familial versus population studies), sample size, ethnic variation, formulation of statistical tests, sample representation, behavior measurement and statistical controls included.

The issue of consistency is essentially an issue of credibility. The recent stringent criteria of p-values and replications set for genome-wide association studies represent a major effort to establish credibility in genetic association studies. In this article, we address the issue of credibility by testing the effect of a *DAT1* genotype across a spectrum of risky behaviors in a single study sample. The analysis has, therefore, neutralized the potential impacts produced by differences in type of studies, sample size, ethnicity, statistical procedures, sample representation and other factors and provided robust and consistent findings often lacking in genetic association studies.

This study tests a specific hypothesis: The 9R/9R genotype in the VNTR of the dopamine transporter gene exerts a general protective effect against a spectrum of risky behaviors in comparison to the 10R/9R and 10R/10R genotypes. The basis of the specific hypothesis is not only the literature on the *DAT1* gene reviewed briefly in this article, but also previously published work that shows specifically that individuals with the 9R/9R genotype had a lower level of delinquency [30] and a smaller number of sexual partners [31] than individuals with the other two genotypes. The study is designed to test if the protective of effect of the *DAT1* gene can be generalized to other risky behaviors.

The central concern for multiple testing is addressed by three features of the study. First, the Add Health genetic dataset contains only a total of five polymorphisms one in each of the five genes, effectively limiting the number of tests that can be performed. Second, the study targets specifically at the protective effect of the 9R/9R genotype in the dopamine transporter gene; this particular hypothesis was generated by previous investigation of delinquency [30] and number of sexual partners [31] in the same Add Health dataset. Three, we obtained evidence for the same genetic effect on a spectrum of most commonly examined risky behaviors.

To test whether the protective effect of the DAT1*9R/9R genotype can be accounted for by background characteristics, we also compare individuals carrying the 9R/9R genotype with individuals carrying the Any10R genotype in terms of age, physical maturity, verbal IQ, GPA, popularity among peers, church attendance, family structure, and parental education.

## Results

### Genetic Main Effects

Table 1 reports behavior and background differences between individuals with the DAT1*9R/9R and DAT1*Any10R genotypes. Two sets of results are reported for each behavior trait and each background characteristic: one set from the sample mean comparison (2^nd^ and 3^rd^ columns in Table 1) and the second set from regression analysis (4^th^ column). The sample size for each genotype is provided in the mean comparison. Table 1 reports the original form of the regression coefficient for the 9R/9R genotype when the regression is linear. To facilitate interpretation, we report the exponentiated regression coefficient (which is odds ratio or count ratio) when the regression is Poisson, logistic, or ordered logistic. A verbal interpretation of these main effects is also provided in Table 1.

**Table 1:** Behavior and background differences between individuals with the *DAT1* * *9R/9R* and *DAT1* * *Any10R* genotypes, white males, and Add Health Waves I–III: Main Effects Models.

	Mean by Genotype (sample size)		GEE Model 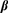 or 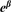 (P-value)	Verbal Interpretation of Effect of 9R/9R	GEE Type
	9R/9R	Any10R	Effect of 9R/9R	Relative to Any10R, those with 9R/9R have	
**RISKY BEHAVIOR**					
Delinquency	0.92(141)	1.60 (2,186)	−0.58(0.033)*	a score 0.58 lower	Lin
# sex partners	1.18 (131)	2.29 (2,058)	0.58†(0.0015)*	42% fewer partners	Poi
Drinking binge	1.49(138)	1.88(2,159)	0.83†(0.093)+	17% fewer binge drink- episodes	Poi
Drinking quantity	2.54(138)	3.76(2,155)	0.69†(0.0092)*	31% fewer drinks when drink	Poi
Smoking quantity	2.33(141)	3.97(2,186)	0.69†(0.14)	31% fewer cigarettes in a day	Poi
Smoking freq	4.49(141)	7.63(2,181)	0.67†(0.068)+	33% fewer smoking days	Poi
Marijuana	1.12(141)	3.06(2,171)	0.382†(0.045)*	62% fewer times of marijuana use	Poi
Cocaine	0.01(141)	0.08(2,184)	0.092†(0.019)*	91% fewer times of cocaine use	Poi
Other illegal drugs	0.03(141)	0.29(2,181)	0.10†(0.0047)**	90% fewer times of other d. use	Poi
Seatbelt wearing	3.22(94)	2.96(1,443)	0.264 (0.093)+	a score 0.26 higher	Lin
**BACKGROUND TRAITS**					
Age	17.91(141)	18.13(2,190)	0.021(0.44)	—	Lin
Physical maturity	3.24(92)	3.21(1,424)	0.025(0.87)	—	Lin
PVT(IQ)	105.8 (90)	103.4(1,447)	2.53(0.04)*		Lin
GPA	2.89(82)	2.73(1,213)	0.17(0.13)	—	Lin
Popularity received	5.34(32)	5.2(534)	−0.427(0.353)	—	Lin
Popularity sent	4.5(32)	4.66(534)	−0.048(0.893)	—	Lin
Church attendance	1.91(140)	1.80(2,169)	0.122†(0.55)	—	Olog
2 bio parents	0.64(94)	0.65(1,443)	1.059†(0.19)	—	Log
Parent education	2.84(44)	2.77(692)	0.882†(0.681)	—	Olog

“*” indicates a statistically significant result at the level of 0.05.

“+” indicates a statistically significant result at the level of 0.10.

^“†”^ indicates that the coefficient is exponentiated (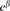).

“Lin, Poi, Olog, and Log” indicate a linear regression, Poisson regression, ordered logit regression, and logit regression models, respectively.

The mean-comparison analysis provides initial support for the hypothesis that individuals with the DAT1*9R/9R genotype are behaviorally more conservative than individuals with the DAT1*Any10R genotype. For every risky behavior we examined, the mean level of risky behavior among individuals with the 9R/9R genotype is lower than the level among those with the Any10R genotype. We illustrate the findings with a number of examples below. The level of delinquency among the 9R/9R genotype (0.92) is about one half of that for the Any10R genotype (1.60). Individuals with the 9R/9R genotype have had an average of 1.49 binge drinking episodes versus 1.88 such episodes for individuals with the Any10R genotype over the past 12 months. Similarly, the 9R/9R individuals on average used marijuana 1.12 times over the past 30 days in comparison to 3.06 times by the Any10R individuals. Also in agreement is the finding for seatbelt-wearing. The 9R/9R adolescents reported greater seatbelt use (3.22) than their Any10R counterparts (2.96).

Participants with the 9R/9R genotype, on average, reported 1.18 sexual partners as compared with 2.29 for the Any10R genotypes. These numbers are averaged over the three Waves at which the measure was reported. The reported number of sexual partners is much smaller at Wave I when the study participants were aged 12–18 than at Wave III when the study participants were aged 18–26. At Wave III, those with 9R/9R reported 2.94 sexual partners as compared with 5.66 for the Any10R genotype.

The GEE regression analysis confirms the findings from the mean-comparison analysis. The GEE regression analysis consists of the main-effect analysis (fourth column in Table 1) and the gene-lifecourse interaction analysis. All regression models estimate the effect of 9R/9R and use Any10R as the reference category. The direction of the 9R/9R effect in all ten main-effect models (Table 1) is consistent with our hypothesis. Six out of the ten estimated main effects are statistically significant at the level of 0.05 or lower. Three out of ten are significant at 0.10 and one has a P-value of 0.14. The lower half of Table 1 presents the estimated differences between the 9R/9R genotype and the Any10R genotype in background characteristics. Out of the ten characteristics, only the verbal IQ differs significantly between the two genotype groups. The difference is small: on average, the 9R/9R genotype scores about 2% higher than the Any10R genotype.

**Figure 1:**
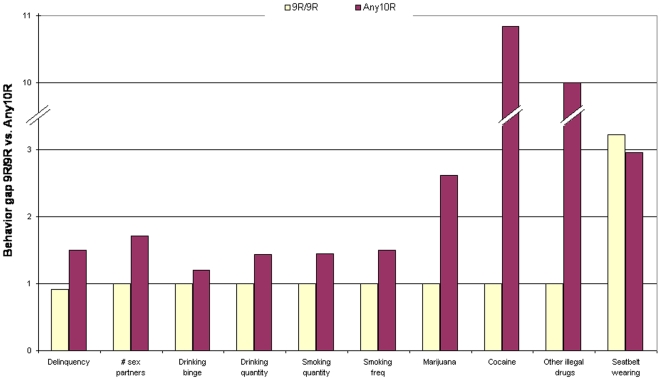
Behavior gap between the DAT1*9R/9R and the DAT1*Any10R genotypes among white males: ten risky behaviors.

**Figure 2:**
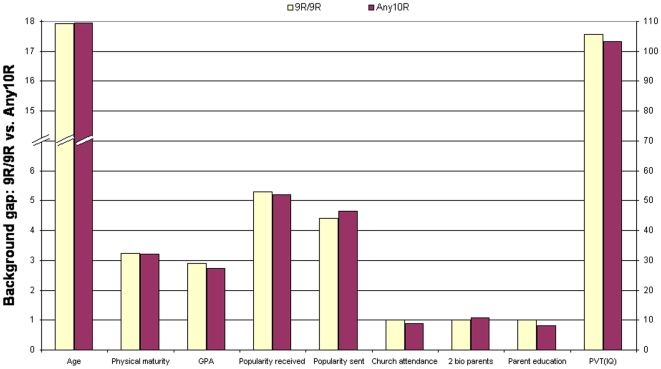
Background gap between the DAT1*9R/9R and the DAT1*Any10R genotypes among white males.

Figure 1 plots the main effect of the Any10R genotype relative to the 9R/9R genotype for the ten risky behaviors. The results for delinquency and seatbelt usage are based on linear regression. For these two behaviors, the estimated level of behavior is plotted for the two genotypes. For example, the levels of delinquency are 1.02 and 1.02+0.58 = 1.60, respectively for the 9R/9R and Any10R genotypes. The difference between 1.02 and 1.60 is statistically significant with a *P*-value of 0.03. For the other eight behaviors, count ratios are plotted. For instance, Figure 1 plots the ratio of the count for the Any10R genotype to the count of the 9R/9R genotype with the count of the 9R/9R genotype set as one. For example, individuals with the Any10R genotype reported about 72% (1/0.58 = 1.72) more sexual partners than the 9R/9R genotype, and the associated *P*-value is 0.0015. The results concerning the background characteristics are graphed in Figure 2.

### Gene-Lifecourse Interaction Effects

Table 2 presents the coefficients and their *t* statistics of the gene-lifecourse interaction models for nine risky behaviors. The interaction model was not estimated for *number of sexual partners* because unlike the other behavior measures, number of partners measures the lifetime cumulative number of partners at each Add Health Wave and is thus inappropriate for gene-lifecourse analysis. The main-effect analysis forces the protective effect to be constant over adolescence and young adulthood, estimating an average effect over the age range of 13–25. If the protective effect is only present in a portion of the age range and not in another, averaging over the effects in both portions may yield an effect which is weaker, less statistically significant, or statistically non-significant. Thus, a non-significant main effect does not necessarily indicate an absence of a protective effect. A gene-lifecourse interaction model tests if a protective effect is only present in a portion of the age range of 13–25.

**Table 2:** Behavior differences between individuals with the *DAT1* * *9R/9R* and *DAT1* * *Any10R* genotypes, white males, and Add Health Waves I–III: Age-Gene Interaction Models.

	Regression coefficient (P-value)		
	Log(age)	9R/9R	Log(age)*9R/9R
**RISKY BEHAVIOR**			
Delinquency	−2.5(<.0001)***	−4.43(0.0307)*	1.31(0.050)+
Drinking binge	1.95(<.0001)***	−4.05(0.015)*	1.30(0.019)*
Drinking quantity	1.08(<.0001)***	−3.83(0.0052)+	1.18(0.069)+
Smoking quantity	2.24(<.0001)***	−8.37(0.0017)**	2.67(0.0025)**
Smoking freq	1.79(<.0001)***	−5.95(0.023)*	1.87(0.031)*
Marijuana	1.94(.0003)***	−10.7(0.0035)**	3.24(0.0067)**
Cocaine	6.04(0.0002)***	−23.4(0.0006)***	6.76(0.0027)**
Other illegal drugs	0.73(0.56)	−18.8(<.0001)***	5.55(<0.0001)***
Seatbelt wearing	0.003(0.99)	−5.88(0.041)*	2.22(0.030)*

“*” indicates a statistically significant result at the level of 0.05.

“+” indicates a statistically significant result at the level of 0.10.

Two discoveries from the interaction analysis (Table 2) are particularly noticeable and relevant. First, almost all of the main effects as well as the interaction effects are statistically significant despite the fact that the interaction analysis costs one additional parameter. More parameters tend to decrease the level of significance, but in this case, the additional parameter increases the overall significance of the model. Second, the four interaction models involving binge drinking, smoking quantity, smoking frequency, and seatbelt wearing are highly significant, which contrasts conspicuously with the four main-effect models involving the same four outcomes in Table 1. In the main-effect models, the effect of 9R/9R is either marginally significant (binge drinking, smoking frequency, and seatbelt wearing) or non-significant (smoking quantity). The two discoveries suggest presence of a gene-lifecourse interaction for the protective effect or that the strength of the protection may indeed depend on age over adolescence and young adulthood.

**Figure 3:**
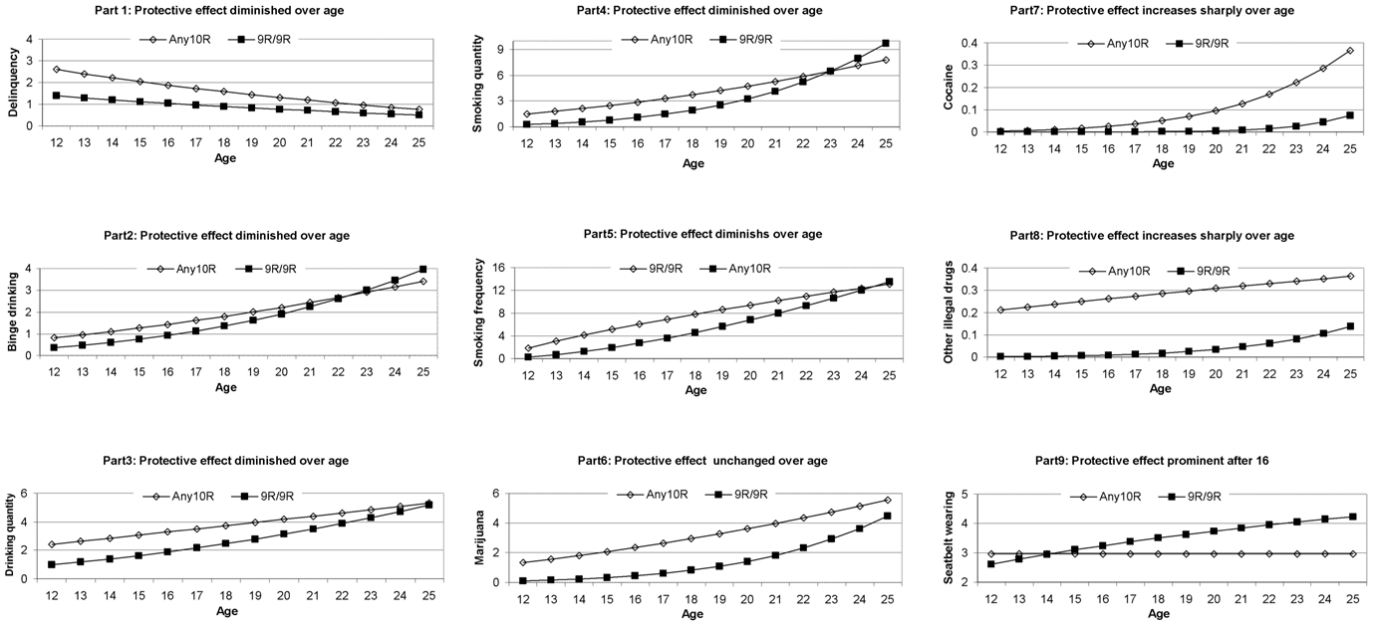
The protective effect of the DAT1*9R/9R genotype relative to the DAT1*Any10R genotype depends on age in adolescence and young adulthood: Parts 1–9.

To interpret the findings of the interaction analysis, we graph the protective effect of the DAT1*9R/9R genotype relative to the DAT1*Any10R genotype as a function of age over adolescence and young adulthood. Figure 3 has 9 parts, one for each behavior measure. The lines stand for the predicted values from regression analysis in Table 2. The lines in Parts 1 and 9, based on linear regression, represent the predicted levels of delinquency and seatbelt wearing, respectively. Parts 2 through 8, based on Passion regression, present the predicted count of a particular behavior. For example, Parts 6–8 plot the number of times that study participants used an illicit drug over the previous 30 days. The prevalence is reflected by both the level of the lines and the unit on the vertical axis. For instance, the prevalence rate of marijuana use is about 10 times as high as cocaine use.

The nine graphs display a small number of patterns of the gene-lifecourse interaction. For binge drinking, drinking quantity, smoking quantity, and smoking frequency, the protective effect of 9R/9R diminishes in young adulthood. In contrast, the protective effect remains large in both adolescence and young adulthood for marijuana use and other illegal drugs and increases sharply for cocaine use. For seatbelt wearing, the protective effect becomes prominent only after the ages of 17–18. In the next section, we show that all of these gene-lifecourse interaction patterns can be explained by a single societal factor.

## Discussion

One intriguing insight from this analysis is that all of the patterns of gene-lifecourse interactions exhibited in Figure 3 can be explained by one single factor: the age-specific legal status of the behaviors or the age-specific social tolerance for the behaviors. The common pattern of lifecourse-gene interaction for binge drinking, drinking quantity, smoking quantity, and smoking frequency can be explained by the legal age for alcohol and smoking. In all of these cases, the protective effect is more prominent during adolescence when drinking and smoking are illegal than during young adulthood when drinking and smoking are becoming legal and more accepted.

The National Minimum Drinking Age Act of 1984 required that all states to raise the legal age for purchase and public possession of alcohol to 21 and tied this to the highway funds [32]. All 50 states in the U.S. attempt to limit youth access to cigarettes by banning sales to individuals younger than 18 or 19 years old [33], [34]. In spite of these alcohol and tobacco laws, under-age drinking and smoking are common in the U.S. [35]. Our empirical evidence shows that underage drinking and smoking do not happen randomly. Individuals with the 9R/9R genotype are less likely to engage in drinking and smoking; however, this protective effect tends to diminish and disappear when drinking and smoking are tolerated as these individuals grow from adolescence into young adulthood. In young adulthood, more legally and socially tolerated drinking and smoking are not considered as risky as in adolescence.

The pattern of gene-lifecourse interaction for illicit drug use (Parts 6–8) can also be explained by the same legal/social factor. Illegal drugs in this study are measured by three variables: *marijuana*, *cocaine*, and *other illegal drugs*. These illicit drugs differ from alcohol and tobacco in at least two important aspects [36]. First, compared with the age-specific legal restrictions for the possession and sale of alcohol and tobacco, these drugs are decidedly illegal and illegal for all ages. Second, the prevalence rates of these drugs are much lower than alcohol and tobacco. Marijuana is by far the most commonly used illicit drug in the United States and worldwide; still, the possession and sale of any quantity of marijuana is prohibited by federal law in all but twelve states. In contrast to drinking and smoking, where the protective effect of 9R/9R quickly diminishes beyond adolescence because of legal and social acceptance of drinking and smoking in young adulthood, the protective effect of 9R/9R for illicit drug use continues beyond adolescence because illicit drug use in young adulthood is no more socially and legally tolerated than in adolescence.

Seatbelt wearing represents a third pattern of gene-lifecourse interaction. The much larger protective effect of 9R/9R after the ages of 15–16 is not accidental; it can be explained by the legal driving age of 16 in the United States. The legal driving age is much more observed than the legal age for alcohol and/or tobacco probably because the access to a car is much harder than alcohol/tobacco and because the perceived consequences of driving a car below the legal age are more severe than using alcohol and tobacco below the legal age. Before the legal driving age, an adolescent is not driving, and his or her friends are likely not driving. An adolescent under the legal driving age is much more likely to be under a supervision of an adult than when he or she is over 16 when he or she would have much greater freedom to decide whether to wear a seatbelt, hence the increased protective effect after age 16.

Delinquency represents a distinct case. The protective effect of 9R/9R against delinquency is most pronounced during the early and mid adolescence; it declines thereafter (Part 1 in Figure 3). The delinquency scale is designed to capture a wide range of serious illegal behaviors that could result in state sanction of arrest, conviction, and incarceration. That delinquency is illegal at all ages suggests a constant protective effect across ages. However, unlike alcohol use, tobacco use, and illicit drug use, which peak in young adulthood, delinquency reaches the highest level in adolescence. The sharp decline of delinquency from adolescence to young adulthood has been observed and documented universally in different cultures and across historical time [37]. Part 1 in Figure 3 shows that the level of delinquency declines sharply for both the Any10R genotype and the 9R/9R genotype, more so for the former than the latter. Our interpretation of the gene-lifecourse interaction is: The universal and dramatic reduction in delinquency beyond adolescence is itself an immense protective factor. The age protection is so large that it renders the protection of 9R/9R less noteworthy.

In summary, our data have revealed two empirical findings: (1) A protective effect related to the *DAT1* gene against risky behaviors is consistently found for delinquency, variety of sexual partners, binge drinking, drinking quantity, smoking quantity, smoking frequency, marijuana use, cocaine use, other illegal drug use, and seatbelt non-wearing; and (2) the strength of the protective effect varies over ages in adolescence and young adulthood, being strong at the ages when the particular behavior is illegal and weakening at the ages when the particular behavior becomes legal or more socially tolerated.

The protective effects exhibit stronger statistical significance in the gene-lifecourse interaction models than in the main effect models because the interaction model allows the protective effect to be more important at some ages than others. The main effect model forces the protective effect to be constant across all ages in adolescence and young adulthood. We also examined the question of whether the individuals in the two genotype groups (9R/9R and Any10R) systematically differ in age, level of physical maturity, verbal IQ, grade point average, received popularity, sent popularity, church attendance, presence of two biological parents in household, and level of parental education. The two genotype groups differ only in one of these background traits: The 9R/9R individuals score about 2% higher on a verbal IQ than the Any10R ones. The lack of differences in background traits between the two genotype groups suggests that the protective effect of 9R/9R cannot be explained away by non-genetic influences at the individual and familial levels.

These two pieces of empirical evidence suggest the protective effect as a general effect concerning risk aversion or behavior conservatism. The evidence does not support the argument that *DAT1* is a gene specifically for craving alcohol or tobacco, an appetite for sexual partner variety, or a propensity for violence for three reasons. First, quite different biochemical mechanisms may be at work for alcohol craving, tobacco addiction, sexual preference, or violent inclination. A single genetic variant in *DAT1* is unlikely to be responsible for all the biochemical processes that underlie such a variety of behaviors. Second, the large majority of white males (94%) belong to the “higher risk” group of the Any10R genotype. Although this group scored higher on all of the ten risky behavior measures investigated in this study, it is more appropriate to view this large majority as representative of the population average. It is the 6% possessing the 9R/9R genotype that stand out as behavioral conservatives or straight arrows. Third, the idea of a general protective effect of the 9R/9R genotype is further strengthened by the findings from the gene-age interactions. The findings show that the protection of the genotype is sensitive to a general level of legal tolerance. These findings generate a further hypothesis that can be tested in future work: It is beyond adolescence when drinking and smoking become legal that behaviors such as drinking and smoking be governed more and more by genes related to specific drug addiction processes.

Dick and colleagues [38] investigated the role of the GABRA2 gene in alcohol and illicit drug dependence across developmental stages using a COGA-based sample. Our findings appear in agreement with theirs. In their sample, the gene is associated with alcohol dependence at ages of 15–20; but this association diminishes in early 20s. GABRA2 also affects illicit drug dependence in their sample in both adolescence and later life stages.

Two goals of genetic association studies are (1) to find credible evidence linking genetic variations to human traits and (2) to understand the contexts of such a link. Major headway has been made recently for the first target via genome-wide association studies (GWAS). A recent succession of GWAS identified genetic variants associated with a number of human diseases [e.g.], [39], [40], [41], [42,43]. GWAS aims at discovering effects of novel genetic variants—effects that are averaged over a large number of individuals. Understanding the context in which a genetic effect operates is not a primary target in GWAS.

Our analysis has made significant headway towards both targets of providing credible evidence and understanding the context of such an effect for genetic association studies. The credibility of evidence is established by demonstrating a similar protective effect across a spectrum of ten risky behavior traits measured on the same set of individuals. Our work has demonstrated how information such as lifecourse of risky behaviors could be used to help illuminate the legal, social, and cultural contexts in which a genetic effect operates.

A number of limitations should be noted. We were able to examine only one VNTR in the *DAT1* gene. Other variants within *DAT1* or elsewhere that are in LD with the VNTR may be the causal behavior-influencing variants. The likelihood is high that some other genetic variants that are not in LD with *DAT1* traits also affect the set of risky health behaviors we have examined. We are not able to examine CNV and epigenetic variations in this study. Finally, although we have replicated our basic results across a large number of risky behaviors, these findings need to be replicated in other independent datasets.

## Methods

### Data Source

The data source for our analysis is the DNA sub-sample of 2,500 siblings in the National Longitudinal Study of Adolescent Health (Add Health), which initiated as a nationally representative sample of about 20,000 adolescents in grades 7–12 from 134 schools in 1994–5 (Wave I) in the United States [44]. The school sample is stratified by region of the country, ethnic mix, size, urbanicity (urban/suburban/rural), and school type (public/private/parochial). Add Health is longitudinal; initial interviews with respondents were followed by two additional in-home interviews in 1996 (Wave II) and 2001–2002 (Wave III). Our analysis uses the sibling sub-sample of Add Health because DNA measures collected at Wave III in 2001–2002 are available only for this subset of the respondents. The subset consists of about 2,500 MZ twins, DZ twins, full biological siblings, and singletons.

The present study is based on about 822 Non-Hispanic white males in Add Health. The sample size varies moderately across behavior traits and Add Health Waves, depending on the extent of missing values. This study focuses on males because males and females often exhibit large differences in the investigated behaviors. For example, female delinquent and criminal participation has been shown to be universally and dramatically lower than that of males [45]. Mice transgenic or knockout studies of aggressive behavior show that genetic influences are often sex-specific [46]. The present study focuses on Caucasians and excludes other ethnicities to reduce the potential impact of population admixture. The role of a gene can differ sharply across ethnic/racial groups. In Add Health, other ethnic groups including Hispanics, African Americans, Asians, and Native Americans jointly account for about 35% of the cohort.

### DNA Preparation and Genotyping

At Wave III in 2002, in collaboration with the Institute for Behavioral Genetics in Boulder, Colorado, Add Health collected, extracted, and quantified DNA samples from the sibling sub-sample. Genomic DNA was isolated from buccal cells using a modification of published methods [47], [48], [49]. The additional details on DNA collection and genotyping are provided at the Add Health website (Smolen and Hewitt, http://www.cpc.unc.edu/projects/addhealth/).

A 40-bp variable number tandem repeat (VNTR) polymorphism in the 3′ untranslated region of the *DAT1* gene has been genotyped with the modified method used by Vandenbergh et al (1992). This VNTR ranges from 3 to 11 copies with the 9-repeat (9R or 440 bp) and 10-repeat (10R or 480 bp) polymorphisms being the two most common alleles [50]. In the Add Health sibling sample, the 9R and 10R account for about 21% and 76% of all alleles, respectively. Our analysis used only individuals with genotypes of one 10R, two 10Rs, and two 9Rs. The individuals with other genotypes (about 2%) are excluded from the analysis.

### Risky Behaviors

This study examines a spectrum of ten risky behaviors (see Introduction for a list). The measures of risky behaviors are intended to capture behavioral patterns rather than accidental, incidental, or isolated events. For example, for alcohol or tobacco use, we are interested in the quantity that is used consistently rather than the timing of the first trial, which can be an isolated event.

Similar to almost all large-scale human studies, Add Health relies on self-reports to measure behaviors. To protect confidentiality, reduce non-responses, and increase reporting accuracy, the interview sections focusing on risky behaviors in Add Health are self-administered by audio-CASI (Computer Assisted Self Interview). A sensitive question is read to respondents from an electronic voice and respondents then confidentially enter their response into a laptop computer. This technique has been shown to increase response rates and reduce biases when sensitive questions are involved [51], [52], [53]. Self reports are now a fundamental method of behavior measuring and seem capable of yielding reliable and valid data [54].

Most of the behaviors we investigate are measured repeatedly on the same study participants across all three Add Health Waves. The study analyzes all the repeated measures and makes corresponding statistical adjustments. Table 3 describes each of the behavior and background variables used in the analysis. The description provides the variable definition; information on how the variable is constructed; the sample mean/proportion and its sample size at each of the three Add Health Waves; and the sample mean/proportion, standard deviation, and sample size of the overall Add Health sample that contains all the repeated measures at Waves I–III. Thus, the sample size of the overall sample (the last column) should be equal to the sum of the three samples from Waves I–III.

**Table 3:** Behavior and background measures: definition, construction, mean(sample size) at each Add Health wave, and overall mean(SD)(sample size) for white males.

			Wave I	Wave II	Wave III	Waves I–III
Age Range			12–19	13–20	18–26	12–26
	Definition	Variable Construction	Mean(N)	Mean(N)	Mean(N)	Mean(SD)(N)
**RISKY BEHAVIOR**						
**Delinquency**	delinquent Behaviors	Weighted average of 12 items	2.25(820)	1.58(766)	1.10(821)	1.65(3.31)(2407)
**Number of sex partners**	With how many partners have you ever had vaginal intercourse, even if only once?	Count variable	0.33(818)	0.36(766)	6.74(670)	2.22(5.42)( 2189)
**Drinking binge**	Over the past 12 months, on how many days did you drink five or more drinks in a row?	7 = daily/almost daily6 = 3 to 5 days a week5 = 1 or 2 days a week4 = 2 or 3 a month3 = <once a month2 = 1 or 2 days past yr1 = never past yr0 = never	1.30(813)	1.62(758)	2.64(804)	1.86(2.02)(2375)
**Drinking quantity**	Think of all the times you have had a drink during the past 12 months. How many drinks did you usually have each time?	Count variable	2.30(810)	3.46(751)	4.43(807)	3.63(5.29)(2368)
**Smoking quantity**	During the past 30 days, on the days you smoked, how many cigarettes did you smoke each day?	Count variable	3.11(679)	3.79(669)	5.89(815)	4.28(7.88)(2163)
**Smoking frequency**	During the past 30 days, on how many days did you smoke cigarettes?	Count variable	5.34(813)	6.62(758)	10.66 (813)	7.56(12.3)(2384)
**Marijuana**	During the past 30 days, how many times did you use marijuana?	Count variable>100 = 100	2.10(814)	2.11(752)	4.57(808)	2.76(9.99)(2374)
**Cocaine**	During the past 30 days, how many times did you use cocaine?	Count variable>100 = 100	0.016(817)	0.041(764)	0.180(812)	0.080(0.941)(2393)
**Other illegal drugs**	During the past 30 days, how many times did you use any of these types of illegal drugs?	Count variable>100 = 100(LSD, PCP, ecstasy, mushrooms, speed, ice, heroin, or pills)	0.362(816)	0.177(761)	0.290(809)	0.274(2.86)(2386)
**Seatbelt wearing**	How often do you wear a seatbelt when you are riding in or driving a car?	0 = never1 = rarely2 = sometimes3 = most often4 = always	2.93(822)	3.00(767)	Not collected	2.97(1.22)(1589)
**BACKGROUND TRAITS**						
**Age**	Age at interview at each wave	Calculated from interview year and month as well as year and month of birth	16.1(822)	16.6(822)	22.0(822)	18.2(2466)
**Physical maturity**	How advanced is your physical development compared to other boys of your age?	1 = younger than most2 = younger than some3 = average4 = older than some5 = older than most	3.27(808)	3.19(759)	Not collected	3.23(1.11)(1567)
**PVT (verbal IQ)**	Peabody Vocabulary Picture Test for Add Health	Longitudinally standardized	103.1(785)	Not collected	104.2(806)	103.6(12.28)(1591)
**GPA English**	At the most recent grading period, what was your grade in English or language arts?	1 = A, 2 = B, 3 = C, and 4 = D or lower	2.73(793)	2.75(657)	Not Collected	2.74(0.966)(1450)
**GPA math**	What was your grade in mathematics?	Same as above	2.72(767)	2.78(597)	Not Collected	2.75(1.05)(1364)
**GPA total**	Average over English and math	Same as above	2.73(757)	2.77(584)	Not Collected	2.75(0.846)(1341)
**Popularity received**	Number of friend nominations received by respondent	Count variable	5.20(583)			5.20(4.11)(583)
**Popularity Sent**	Number of friends nominated by respondent	Count variable	4.64(583)			4.64(3.10)(583)
**Church attendance**	In the past 12 months, how often did you attend religious services?	0 = never;1<once a month2 = >once a month3 = >once a weekTreated as an ordered variable	1.65(809)	1.54(760)	1.15(820)	1.44(1.19)(796)
**Two biological Parents**	Constructed from information family structure	Binary variable	0.669(818)	0.627(766)	Not collected	0.65(0.48)(1584)
**Parental education**	How far in school did your biological father go? How far in school did your biological mother go?	1<high school2 = high school3<less than college4 = >collegeCoded as an ordered variable (the higher of the two)	3.08(802)	Not used	Not used	3.08(0.941)(802)

*Delinquency* measures delinquent behaviors including violent behaviors among adolescents and young adults. The construct is based on 12 questions asked of all the Add Health respondents at Waves I–III. The questions and scaling weights used to create the scale can be found in Guo et al. [55]. The delinquency scale is constructed using information on stealing amounts larger or smaller than $50, breaking and entering, selling drugs, serious physical fighting that resulted in injuries needing medical treatment, use of weapons to get something from someone, involvement in physical fighting between groups, shooting or stabbing someone, deliberately damaging property, and pulling a knife or gun on someone. Table 3 indicates that 820, 766, and 821 white male adolescents and young adults contribute a measure of delinquency at Waves I, II, and III, respectively. Our final analysis file consists of 2,407 person-measures of delinquency. The delinquency scale is a variation of a widely-used type of scales in contemporary research on delinquency and criminal behavior [54].

*Number of Sexual Partners* is based on the answer to the question of “With how many partners have you ever had vaginal intercourse, even if only once?” The question was repeated at Waves I–III. Two alcohol-related measures are investigated in this analysis: *binge drinking* and *drinking quantity*. Both are measured three times in Add Health. *Binge drinking* is constructed from the question “Over the past 12 months, on how many days did you drink five or more drinks in a row?” *Drinking quantity* measures the typical number of drinks a respondent consumes each time he or she drinks.

*Smoking quantity* is a measure of number of cigarettes a respondent smokes on the days he or she smokes. *Smoking frequency* records the number of days a respondent smoked cigarettes over the past 30 days. The three variables of illegal drug use (*marijuana*, *cocaine*, and *other illegal drugs*) measure the number of times a respondent has used the drug(s) over the past 30 days. Other illegal drugs refer to LSD, PCP, ecstasy, mushrooms, speed, ice, heroin, and/or pills. The two smoking measures and the three measures of illegal drug use are all collected at Add Health Waves I–III. *Seatbelt wearing* is included in the analysis because not wearing a seatbelt represents a risky behavior. The variable is based on the question of how often a respondent wears a seatbelt when riding in or driving a motor vehicle. The question was asked at Add Health Waves I–II.

### Background Characteristics

To provide further evidence that the differences in the ten behaviors are a result of the variations in the *DAT1* VNTR rather than the differences in other individual and socioeconomic characteristics, we examined the differences in an array of background characteristics between those with the 9R/9R genotype and those with the Any10R genotype. The following section briefly describes the construction of these background variables. *Age* is calculated from interview year and month as well as year and month of birth. To protect participants' confidentiality, Add Health does not disclose interview day and birth day of a participant. *Physical maturity* is from the self-assessment of physical development compared to other boys of same age.

*Verbal IQ* or the Add Health *Picture Vocabulary Test* (AHPVT) is a slightly shortened version of the standard Peabody Picture Vocabulary Test [56], [57], which is usually considered a verbal IQ test. *GPA*, grade point average, is an average of self-reported grades in English and mathematics. One of the main innovations of the Add Health study is in the area of social network data collection. In the in-school study at Wave I, a brief interview was administered to all students in a school, in which Add Health participants were asked to nominate up to 5 same-sex and 5 opposite-sex friends starting from the best friend. *Popularity received*, or the social prestige of an adolescent, is measured by the number of times the respondent was nominated by other students in school. *Popularity sent* is measured by the number of friends the respondent nominated.

*Church attendance* is created from the self-reported frequency at which a study participant attended religious services in the past 12 months. *Presence of two biological parents* in the household at the time of interview is constructed from information on family structure at each Add Health Wave. *Parental education* records the higher level of education of father and mother.

### Statistical Methods

Our analytical method consists of two steps: a mean or proportion comparison and a regression analysis. The mean-comparison analysis compares the levels of risky behaviors between the 9R/9R genotype and the Any10R genotype. No statistical test is carried out in the mean comparison analysis because standard tests are not valid due to the correlations among the observations from sibling clustering and repeated measures of the same individuals at Add Health Waves.

The regression analysis uses generalized estimating equation (GEE) models to estimate the association between the *DAT1* VNTR and risky behaviors [58]. The GEE analysis can be viewed as a statistically appropriate mean comparison. It addresses the correlations among the siblings in the dataset, adjusts for age, and carries out the comparison. GEE models have long been established in the statistical literature as a standard approach for addressing correlated data. Our outcome variables for regression analysis have different statistical distributions. For example, *age* and *PVT* are approximately normal; *number of sexual partners* is Poisson; *church attendance* is an ordered categorical variable, and *presence of two biological parents* is binary. All of these outcome variables can be accommodated readily in the GEE modeling framework.

The following equation describes the general form of the GEE model in our analysis:
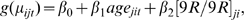
(1)Where *i*, *j*, and *t* index individual, sibling cluster, and Add Health Wave, respectively; 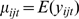; 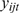 is an observed risky behavior or background characteristic; 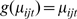 when 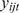 is a continuous outcome; 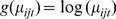 when 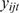 is a count variable; and 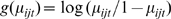 when 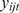 is a binary outcome variable. The correlation structure in the sample is addressed by a three-level GEE model with level 1 for Add Health Wave or repeated measures of the same individual, level 2 for individuals, and level 3 for sibling clusters.

To test whether the protective effect of the 9R/9R genotype interacts with lifecourse, we include a gene-age interaction term in the model
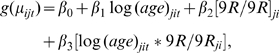
(2)where *age* is modeled on a logarithm scale for two reasons. *Log(age)* can capture a non-linear effect and with one parameter, *Log(age)* is more parsimonious than the more usual alternative *age* and *age^2^*. The gene-lifecourse interaction patterns will be illustrated by graphing.

Our analysis sample includes only white males, thus eliminating the concern for population admixture at the level of race/ethnicity. The white males were drawn from 134 schools across the US and are representative of all white males in the country. Missing data were addressed by case-wise deletion rather than imputation. For example, the number of observations across the three Add Health waves is not the same (Table 3), that is, not all individuals were interviewed at all three waves. Our analysis employed all available observations at all waves without imputing the missing observations.

### Institutional Review Board and Consent of Respondents

The Add Health study was reviewed and approved by the IRB at the University of North Carolina-Chapel Hill before each wave of data collection. In addition, Add Health obtained written consent at all waves from either the respondent or the parent if the respondent was under age 18. Regarding the consent for genotyping, Wave III respondents were informed that “Your DNA will be used to learn how closely you are related to your brother or sister and to study the influence of your genetic makeup on your mind, body, and behavior. Specimens will be stored for as long as they are usable and used only for these research purposes.”

## References

[1] Munafo MR, Clark TG, Johnstone EC, Murphy MFG, Walton RT (2004). The genetic basis for smoking behavior: A systematic review and meta-analysis.. Nicotine & Tobacco Research.

[2] Sieminska A, Buczkowski K, Jassem E, Niedoszytko M, Tkacz E (2009). Influences of polymorphic variants of DRD2 and SLC6A3 genes, and their combinations on smoking in Polish population.. Bmc Medical Genetics.

[3] Bannon MJ, Whitty CJ (1995). NEUROKININ RECEPTOR GENE-EXPRESSION IN SUBSTANTIA-NIGRA - LOCALIZATION, REGULATION, AND POTENTIAL PHYSIOLOGICAL SIGNIFICANCE.. Canadian Journal of Physiology and Pharmacology.

[4] Bressan RA, Crippa JA (2005). The role of dopamine in reward and pleasure behaviour - review of data from preclinical research.. Acta Psychiatrica Scandinavica.

[5] Cragg SJ, Rice ME (2004). DAncing past the DAT at a DA synapse.. Trends in Neurosciences.

[6] Kalivas P, Davis KLCD, Coyle JT, Nemeroff C (2002). Neurocircuitry of addiction.. Neuropsychopharmacology-the fifth generation of progress.

[7] Vandenbergh DJ, Persico AM, Hawkins AL, Griffin CA, Li X (1992). HUMAN DOPAMINE TRANSPORTER GENE (DAT1) MAPS TO CHROMOSOME-5P15.3 AND DISPLAYS A VNTR.. Genomics.

[8] Haddley K, Vasiliou AS, Ali FR, Paredes UM, Bubb VJ (2008). Molecular genetics of monoamine transporters: Relevance to brain disorders.. Neurochemical Research.

[9] Heinz A, Goldman D, Jones DW, Palmour R, Hommer D (2000). Genotype influences in vivo dopamine transporter availability in human striatum.. Neuropsychopharmacology.

[10] Jacobsen LK, Staley JK, Zoghbi S, Seibyl JP, Kosten TR (2000). Prediction of dopamine transporter binding availability by genotype: A preliminary report.. American Journal of Psychiatry.

[11] van Dyck CH, Malison RT, Jacobsen LK, Seibyl JP, Staley JK (2005). Increased dopamine transporter availability associated with the 9-repeat allele of the SLC6A3 gene.. Journal of Nuclear Medicine.

[12] Michelhaugh SK, Fiskerstrand C, Lovejoy E, Bannon MJ, Quinn JP (2001). The dopamine transporter gene (SLC6A3) variable number of tandem repeats domain enhances transcription in dopamine neurons.. Journal of Neurochemistry.

[13] Martinez D, Gelernter J, Abi-Dargham A, van Dyck CH, Kegeles L (2001). The variable number of tandem repeats polymorphism of the dopamine transporter gene is not associated with significant change in dopamine transporter phenotype in humans.. Neuropsychopharmacology.

[14] Lynch DR, Mozley PD, Sokol S, Maas NMC, Balcer LJ (2003). Lack of effect of polymorphisms in dopamine metabolism related genes on imaging of TRODAT-1 in striatum of asymptomatic volunteers and patients with Parkinson's disease.. Movement Disorders.

[15] Sabol SZ, Nelson ML, Fisher C, Gunzerath L, Brody CL (1999). A genetic association for cigarette smoking behavior.. Health Psychology.

[16] Lerman C, Caporaso NE, Audrain J, Main D, Bowman ED (1999). Evidence suggesting the role of specific genetic factors in cigarette smoking.. Health Psychology.

[17] Timberlake DS, Haberstick BC, Lessem JM, Smolen A, Ehringer M (2006). An association between the DAT1 polymorphism and smoking behavior in young adults from the national longitudinal study of adolescent health.. Health Psychology.

[18] Sander T, Harms H, Dufeu P, Kuhn S, Rommelspacher H (1997). Dopamine D4 receptor exon III alleles and variation of novelty seeking in alcoholics.. American Journal of Medical Genetics.

[19] Schmidt LG, Harms H, Kuhn S, Rommelspacher H, Sander T (1998). Modification of alcohol withdrawal by the A(9) allele of the dopamine transporter gene.. American Journal of Psychiatry.

[20] Cook EH, Stein MA, Krasowski MD, Cox NJ, Olkon DM (1995). ASSOCIATION OF ATTENTION-DEFICIT DISORDER AND THE DOPAMINE TRANSPORTER GENE.. American Journal of Human Genetics.

[21] Gill M, Daly G, Heron S, Hawi Z, Fitzgerald M (1997). Confirmation of association between attention deficit hyperactivity disorder and a dopamine transporter polymorphism.. Molecular Psychiatry.

[22] Daly G, Hawi Z, Fitzgerald M, Gill M (1999). Mapping susceptibility loci in attention deficit hyperactivity disorder: preferential transmission of parental alleles at DAT1, DBH and DRD5 to affected children.. Molecular Psychiatry.

[23] Barr CL, Xu C, Kroft J, Feng Y, Wigg K (2001). Haplotype study of three polymorphisms at the dopamine transporter locus confirm linkage to attention-deficit/hyperactivity disorder.. Biological Psychiatry.

[24] Curran S, Mill J, Tahir E, Kent L, Richards S (2001). Association study of a dopamine transporter polymorphism and attention deficit hyperactivity disorder in UK and Turkish samples.. Molecular Psychiatry.

[25] Chen CK, Chen SL, Mill J, Huang YS, Lin SK (2003). The dopamine transporter gene is associated with attention deficit hyperactivity disorder in a Taiwanese sample.. Molecular Psychiatry.

[26] Barkley RA, Smith KM, Fischer M, Navia B (2006). An examination of the behavioral and neuropsychological correlates of three ADHD candidate gene polymorphisms (DRD4 7+, DBH TaqI A2, and DAT1 40 bp VNTR) in hyperactive and normal children followed to adulthood.. American Journal of Medical Genetics Part B-Neuropsychiatric Genetics.

[27] Feng Y, Wigg KG, Makkar R, Ickowicz A, Pathare T (2005). Sequence variation in the 3′-untranslated region of the dopamine transporter gene and attention-deficit hyperactivity disorder (ADHD).. American Journal of Medical Genetics Part B-Neuropsychiatric Genetics.

[28] Swanson JM, Flodman P, Kennedy S, Spence MA, Moyzis R (2000). Dopamine genes and ADHD.. Neuroscience and Biobehavioral Reviews.

[29] Cheuk DKL, Li SYH, Wong V (2006). No association between VNTR polymorphisms of dopamine transporter gene and attention deficit hyperactivity disorder in Chinese children.. American Journal of Medical Genetics Part B-Neuropsychiatric Genetics.

[30] Guo G, Roettger M, Shih JC (2007). Contributions of the DAT1 and DRD2 genes to serious and violent delinquency among adolescents and young adults.. Human Genetics.

[31] Guo G, Tong YY, Xie CW, Lange LA (2007). Dopamine transporter, gender, and number of sexual partners among young adults.. European Journal of Human Genetics.

[32] Mooney LA, Gramling R, Forsyth C (1992). Legal Drinking Age and Alcohol-Consumption.. Deviant Behavior.

[33] Ahmad S (2005). Closing the youth access gap: The projected health benefits and cost savings of a national policy to raise the legal smoking age to 21 in the United States.. Health Policy.

[34] Ahmad S, Billimek J (2007). Limiting youth access to tobacco: Comparing the long-term health impacts of increasing cigarette excise taxes and raising the legal smoking age to 21 in the United States.. Health Policy.

[35] Johnston LD, O'Malley PM, Bachman JG (2003). Monitoring the Future National Survey Results on Drug Use, 1975–2002: Vol. 1. Secondary School Students.

[36] Goode E (2008). Drugs in American Society.

[37] Hirschi T (1969). Causes of Delinquency.

[38] Dick DM, Bierut L, Hinrichs A, Fox L, Bucholz KK (2006). The role of GABRA2 in risk for conduct disorder and alcohol and drug dependence across developmental stages.. Behavior Genetics.

[39] Frayling TM, Timpson NJ, Weedon MN, Zeggini E, Freathy RM (2007). A common variant in the FTO gene is associated with body mass index and predisposes to childhood and adult obesity.. Science.

[40] Zeggini E, Weedon MN, Lindgren CM, Frayling TM, Elliott KS (2007). Replication of genome-wide association signals in UK samples reveals risk loci for type 2 diabetes.. Science.

[41] Scott LJ, Mohlke KL, Bonnycastle LL, Willer CJ, Li Y (2007). A genome-wide association study of type 2 diabetes in Finns detects multiple susceptibility variants.. Science.

[42] Steinthorsdottir V, Thorleifsson G, Reynisdottir I, Benediktsson R, Jonsdottir T (2007). A variant in CDKAL1 influences insulin response and risk of type 2 diabetes.. Nature Genetics.

[43] Sladek R, Rocheleau G, Rung J, Dina C, Shen L (2007). A genome-wide association study identifies novel risk loci for type 2 diabetes.. Nature.

[44] Harris KM, Florey F, Tabor J, Bearman PS, Jones J (2003). The National Longitudinal Study of Adolescent Health: Research design.”. http://www.cpc.unc.edu/projects/addhealth/design.

[45] Gottfredson MR, Hirschi T (1990). A general theory of crime.

[46] Maxson S, Kim KY (In press). The genetics of offensive aggression in mice.. Handbook of Behavior Genetics.

[47] Meulenbelt I, Droog S, Trommelen GJM, Boomsma DI, Slagboom PE (1995). HIGH-YIELD NONINVASIVE HUMAN GENOMIC DNA ISOLATION METHOD FOR GENETIC-STUDIES IN GEOGRAPHICALLY DISPERSED FAMILIES AND POPULATIONS.. American Journal of Human Genetics.

[48] Freeman B, Powell J, Ball D, Hill L, Craig I (1997). DNA by mail: An inexpensive and noninvasive method for collecting DNA samples from widely dispersed populations.. Behavior Genetics.

[49] Lench N, Stanier P, Williamson R (1988). SIMPLE NON-INVASIVE METHOD TO OBTAIN DNA FOR GENE ANALYSIS.. Lancet.

[50] Doucettestamm LA, Blakely DJ, Tian J, Mockus S, Mao J (1995). POPULATION GENETIC-STUDY OF THE HUMAN DOPAMINE TRANSPORTER GENE (DAT1).. Genetic Epidemiology.

[51] Newman JC, Jarlais D, Turner CF, Gribble J, Cooley P (2002). The differential effects of face-to-face and computer interview modes.. American Journal of Public Health.

[52] Des Jarlais DC, Paone D, Milliken J, Turner CF, Miller H (1999). Audio-computer interviewing to measure risk behaviour for HIV among injecting drug users: a quasi-randomised trial.. Lancet.

[53] Turner CF, Ku L, Rogers SM, Lindberg LD, Pleck JH (1998). Adolescent sexual behavior, drug use, and violence: Increased reporting with computer survey technology.. Science.

[54] Thornberry TP, Krohn MD (2000). The self-report method for measuring delinquency and crime. Criminal Justice 2000.

[55] Guo G, Roettger EM, Cai T (2008). The Integration of Genetic Propensities into Social Control Models of Delinquency and Violence among Male Youths.. American Sociological Review.

[56] Lubin B, Larsen RM, Matarazzo JD (1984). PATTERNS OF PSYCHOLOGICAL TEST USAGE IN THE UNITED-STATES - 1935–1982.. American Psychologist.

[57] Rice JA, Brown LF (1967). VALIDITY OF PEABODY PICTURE VOCABULARY TEST IN A SAMPLE OF LOW IQ CHILDREN.. American Journal of Mental Deficiency.

[58] Liang KY, Zeger SL (1993). REGRESSION-ANALYSIS FOR CORRELATED DATA.. Annual Review of Public Health.

